# The m.3291T>C mt-tRNA^Leu(UUR)^ mutation is definitely pathogenic and causes multisystem mitochondrial disease

**DOI:** 10.1016/j.jns.2012.12.003

**Published:** 2013-02-15

**Authors:** John W. Yarham, Emma L. Blakely, Charlotte L. Alston, Mark E. Roberts, John Ealing, Piyali Pal, Douglass M. Turnbull, Robert McFarland, Robert W. Taylor

**Affiliations:** a Wellcome Trust Centre for Mitochondrial Research, Institute for Ageing and Health, Newcastle University, Newcastle upon Tyne, United Kingdom; b Salford Royal NHS Foundation Trust, Greater Manchester Neuroscience Centre, Department of Neurology, Salford, Lanchester, M6 8HD, United Kingdom

**Keywords:** Mitochondrial DNA disease, Lipoma, mt-tRNA^Leu(UUR)^, Evolutionary conservation, Single-fibre studies, Pathogenicity

## Abstract

Mitochondrial tRNA point mutations are important causes of human disease, and have been associated with a diverse range of clinical phenotypes. Definitively proving the pathogenicity of any given mt-tRNA mutation requires combined molecular, genetic and functional studies. Subsequent evaluation of the mutation using a pathogenicity scoring system is often very helpful in concluding whether or not the mutation is causing disease. Despite several independent reports linking the m.3291T>C mutation to disease in humans, albeit in association with several different phenotypes, its pathogenicity remains controversial. A lack of conclusive functional evidence and an over-emphasis on the poor evolutionary conservation of the affected nucleotide have contributed to this controversy. Here we describe an adult patient who presented with deafness and lipomas and evidence of mitochondrial abnormalities in his muscle biopsy, who harbours the m.3291T > C mutation, providing conclusive evidence of pathogenicity through analysis of mutation segregation with cytochrome *c* oxidase (COX) deficiency in single muscle fibres, underlining the importance of performing functional studies when assessing pathogenicity.

## 1 Introduction

Mitochondrial (mt-) tRNA point mutations account for ~ 50% of all pathogenic mtDNA mutations despite occupying < 10% of the mitochondrial genome, emphasising their importance in human pathogenesis [1]. Point mutations in the mt-tRNAs have been linked to a host of mitochondrial disorders and disease phenotypes, with a commonly poor correlation to genotype. Although point mutations have been identified in each of the 22 mt-tRNAs, *MTTL1* – the gene encoding for the mt-tRNA^Leu(UUR)^ – is a particularly well-known hotspot for pathogenic point mutations; most notably the m.3243A > G and m.3271T > C mutations which are linked to the mitochondrial encephalomyopathy with lactic acidosis and stroke-like episode (MELAS) syndrome [2–4].

A heteroplasmic m.3291T>C variant in the *MTTL1* gene has previously been reported on 4 separate occasions, in association with MELAS [2], isolated mild myopathy [5], dementia with hearing loss [6] and cerebellar ataxia with ophthalmoparesis, hearing loss and myopathy [7]. These reports provided strong evidence linking m.3291T>C to disease through genetic analysis and molecular investigations. Crucially however, all four reports lacked functional evidence of pathogenicity from either *trans*mitochondrial cybrid or single muscle fibre studies. As a consequence of this lack of functional data, a recent publication questioned the description of m.3291T>C as ‘pathogenic’ [8]. The primary concern of this report was that the mutation affects a nucleotide position that exhibits poor conservation throughout evolution, and that only a slight change in the minimum free energy structure of mt-tRNA^Leu(UUR)^ would be predicted. Here we describe another patient with the m.3291T > C mutation, who presented with bilateral sensorineural deafness, and through the study of his muscle biopsy, are able to confirm the pathogenicity of this mt-tRNA mutation.

## 2 Case report

A 55 year old male former bus driver with a history of deafness was referred to Neurology with a 4-year history of falls, speech disturbance and weight loss. His only medication was Gliclazide for diabetes mellitus that had been diagnosed two years previously. He had bilateral sensorineural deafness for 15 years prior to his neurological presentation for which no explanation had been given, but hearing aids were of benefit. He neither smoked nor drank alcohol. He was unaware of any family history of a similar disorder.

**Fig. 1:**
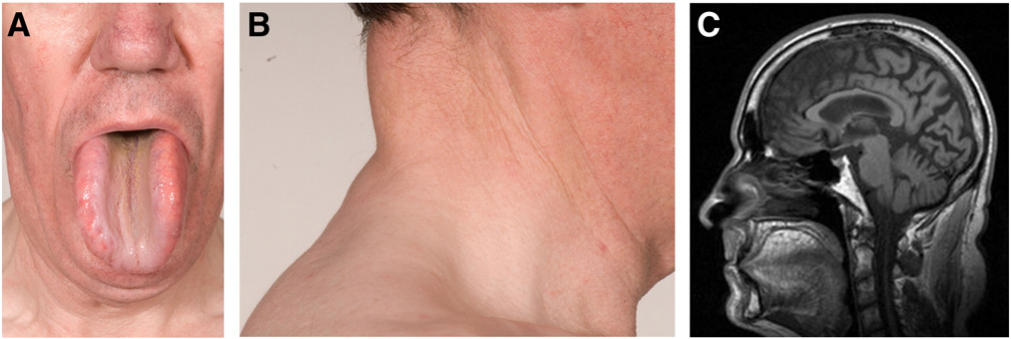
Clinical assessment. The patient was found to have macroglossia (*A*) with a fatty infiltration as well as a prominent dorsocervical fat pad (*B*). Cranial sagittal MRI showed generalised atrophy of the brain and the fatty infiltration of the tongue suggestive of a lipoma (*C*).

Examination revealed macroglossia (Fig. 1A) with associated dysarthria, bilateral hearing aids and bilateral pes cavus. He had a prominent dorsocervical fat pad (Fig. 1B) and a lipoma on his posterior right thigh with an otherwise thin habitus. Muscle bulk was reduced but without focal wasting or fasciculation. Tone was normal but there was a global mild reduction in power, most marked proximally. Reflexes were suppressed in both upper and lower limbs and plantar responses were flexor. Sensory testing revealed reduced dorsal column sensation at the toes and ankles. He had dysmetria of upper and lower limbs with a broad based gait. He was unable to perform a tandem gait. Romberg's testing was normal.

Routine blood tests were unremarkable. Cranial MR imaging showed generalised brain atrophy and fatty infiltration of the tongue (Fig. 1C). CSF was acellular with normal protein and glucose but a raised CSF lactate of 3.7 mmol/L (normal range, 1.1–2.4 mmol/L). Very long chain fatty acids, plasma (including CK) and white cell enzymes were all normal.

Neurophysiology revealed active denervation in all muscles in the lower limbs and the right bicep. Those muscles not showing active denervation demonstrated significantly reduced recruitment. There were no changes to support a myopathic or a large fibre neuropathic process. A tongue biopsy failed to show any evidence of amyloidosis but confirmed fatty infiltration consistent with a lipoma. A clinical diagnosis of mitochondrial disease was made on the basis of a multisystem disorder involving diabetes, deafness, myopathy, ataxia, mid-line lipomata and raised CSF lactate. A right tibialis muscle biopsy was performed under local anaesthetic.

## 3 Materials and methods

### 3.1 Muscle histology and histochemistry

Standard histological (H&E, modified Gomori trichrome staining) and histochemical (cytochrome *c* oxidase (COX), succinate dehydrogenase (SDH) and sequential COX/SDH) analyses of the patient's muscle biopsy were performed on fresh-frozen skeletal muscle sections (10 μm), according to established protocols [9].

### 3.2 Molecular genetic studies

Total DNA was extracted from the patient's whole skeletal muscle and individual (COX-positive and COX-deficient) skeletal muscle fibres isolated by laser microcapture as described previously [10], as well as his asymptomatic sister's blood and urine. Large-scale mtDNA rearrangements were excluded by long-range PCR [11] before sequencing of the entire mitochondrial genome was performed using an ABI 3130xl (Applied Biosystems) system essentially as described elsewhere [12,13].

### 3.3 Assessment of m.3291T>C mutation load by quantitative pyrosequencing

Assessment of mutation load in both whole tissue DNA and DNA lysates from individual skeletal muscle fibres was performed by quantitative pyrosequencing. Pyromark Assay Design Software v.2.0 (Qiagen) was used to design locus specific PCR and pyrosequencing primers, which amplified a 130 bp PCR product spanning the 3291 nucleotide using a biotinylated forward primer (nt 3224-3245): Bio-5′ GGGTTTGTTAAGATGGCAGAGC 3′ and a reverse primer (nt 3353-3330): 5′ GCGATTAGAATGGGTACAATGAGG 3′.

Pyrosequencing was achieved on the Pyromark Q24 platform according to the manufacturer's protocol, employing a mutation-specific pyrosequencing primer (nt 3312-3293): 5′ GGGTATGTTGTTAAGAAGAG 3′. Pyromark Q24 software was used to quantify the m.3291T>C heteroplasmy levels by directly comparing the relevant peak heights of both wild type and mutant mtDNA at this site [14].

## 4 Results

### 4.1 Histology and histochemistry

**Fig. 2:**
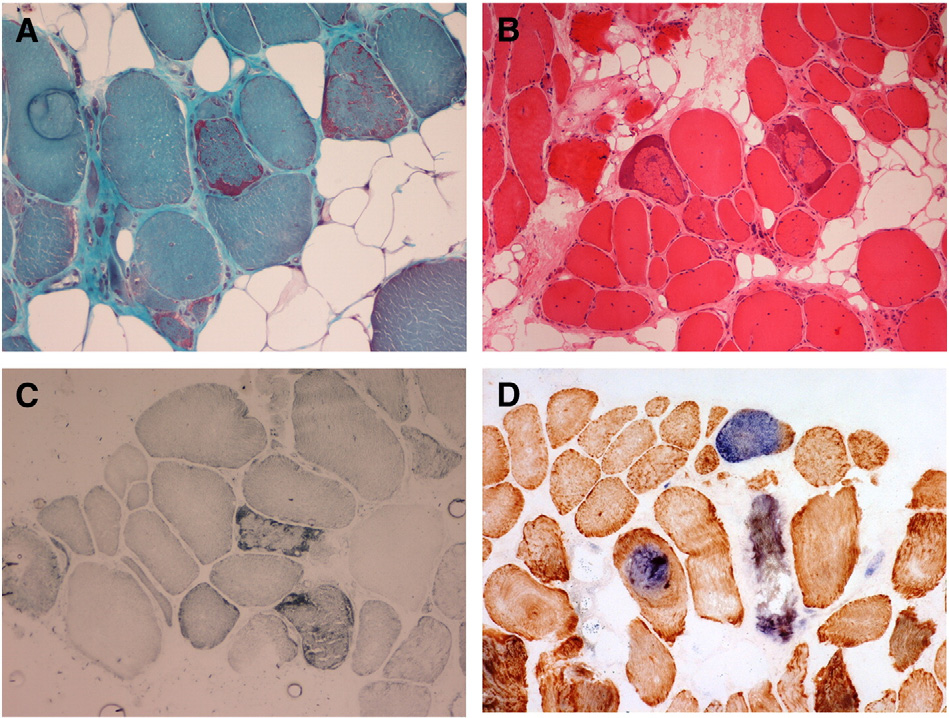
Histological and histochemical analyses of the patient's skeletal muscle biopsy. Both modified Gomori trichrome (*A*) and joint haemotoxylin and eosin (H&E) stain (*B*) of the patient's skeletal muscle showed a dystrophic biopsy with fatty infiltrate and sub-sarcolemmal accumulation of mitochondria. COX-deficient fibres were identified by the individual cytochrome *c* oxidase (COX) reaction (*C*) and the individual succinate dehydrogenase (SDH) reaction (*D*), whilst sequential COX/SDH histochemistry confirmed the identity of true COX-deficient fibres (*D*).

Histological analyses including modified Gomori trichrome (Fig. 2A) and H&E (Fig. 2B) staining revealed a myopathic picture consisting of dystrophic changes, marked fatty infiltrates and fibre replacement. Subsarcolemmal aggregates of mitochondria, typical of “ragged-red” fibres, were also observed, and confirmed by SDH reaction (Fig. 2C). The individual COX reaction showed evidence of COX-deficient fibres that were more clearly identified by the sequential COX/SDH reaction (Fig. 2D). Interestingly, some fibres appear to show only a partial deficiency, with different regions displaying normal COX activity.

### 4.2 Molecular genetic investigations

**Fig. 3:**
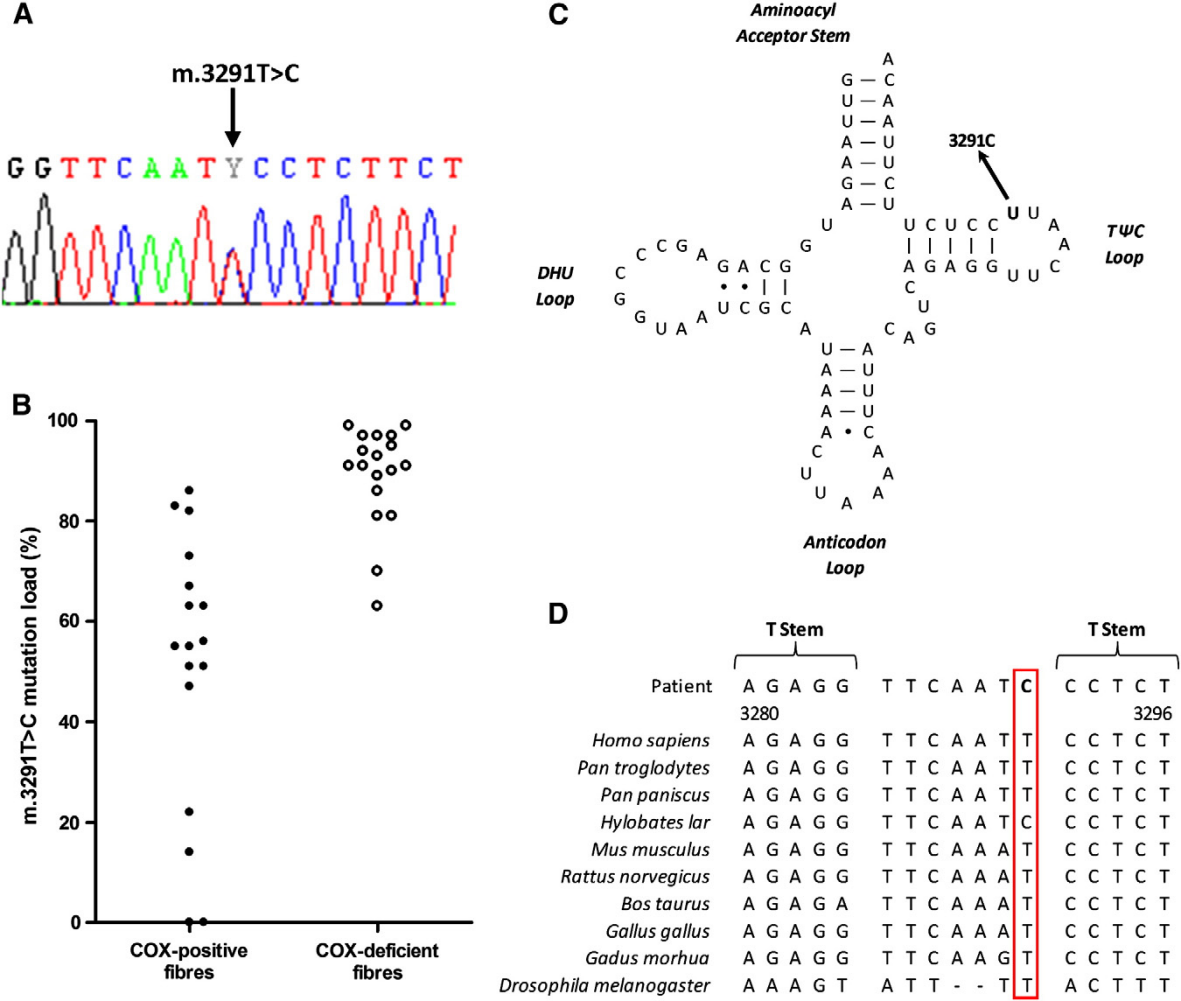
Molecular, genetic and functional investigation of the m.3291T>C mutation. The identified m.3291T>C mutation is shown in a sequencing chromatogram (*A*), whilst mutation load analysis in single COX-positive and COX-deficient fibres demonstrated segregation of the mutation with biochemical deficiency (*B*). The m.3291T>C mutation is located in the T-loop of mt-tRNA^Leu(UUR)^ (*C*), whilst the affected base shows moderate evolutionary conservation across a range of species (*D*).

Large-scale rearrangements of the mtDNA genome were excluded through long-range PCR analysis. Sequencing of the whole mitochondrial genome identified a previously reported m.3291T>C mutation in the *MTTL1* gene which was clearly heteroplasmic (Fig. 3A). Haplogroup analysis placed this patient into haplogroup K1b1a1, with which the m.3291T>C mutation has not been associated, whilst searching of the MitoMap (www.mitomap.org) and mtDB (www.mtdb.igp.uu.se/) databases, and a literature search using PubMed (www.ncbi.nlm.nih.gov/pubmed), confirmed that it is not a recognised polymorphic variant [15,1].

The m.3291T>C mutation was shown by pyrosequencing to be present at heteroplasmic levels in the patient's skeletal muscle (39% mutation load), and at low levels in both blood (6% mutation load) and urine (6% mutation load) from his clinically-unaffected sister, suggestive of maternal transmission.

### 4.3 Single-fibre segregation studies of the m.3291T>C mutation

Single-fibre pyrosequencing was performed on individual COX-positive and COX-deficient fibres to investigate whether the m.3291T>C mutation segregated with respiratory chain deficiency. Only completely COX-deficient and COX-positive fibres were selected, those exhibiting partial deficiency were excluded. The mutation was found at significantly higher levels in COX-deficient fibres (89.1 ± 9.9%, *n* = 18) compared to COX-positive fibres (51.1 ± 27.1%, *n* = 17), a statistically significant finding (P < 0.001, two-tailed Student's *t* test) (Fig. 3B).

## 5 Discussion

Here we describe a patient who presented with neck and tongue lipomas and sensorineural deafness, in whom a previously reported m.3291T>C mutation in mt-tRNA^Leu(UUR)^ was identified. This heteroplasmic substitution has previously been linked to a number of disease presentations, but until now, there has been insufficient functional evidence to confirm its pathogenicity. The single-fibre data we have presented here confirms the pathogenicity of this mutation. This report also reaffirms the heterogeneity of the genotype:phenotype relationship of mt-tRNA point mutations, through the association of m.3291T>C with a new disease phenotype.

The gene for mt-tRNA^Leu(UUR)^, *MTTL1*, is widely acknowledged as a hotspot for mitochondrial disease-causing point mutations and is most commonly associated with the MELAS syndrome. Interestingly, the patient here did not have MELAS, but rather bilateral deafness and fatty deposits resembling lipomas. Previously, lipomas have been associated primarily with the m.8344A>G mutation in mt-tRNA^Lys^ [16–18], although the m.3271T>C mutation has also been linked to lipoma formation [19].

A skeletal muscle biopsy from the patient was found to be highly dystrophic, with considerable fatty deposits, ragged-red fibres and a low level of COX-deficient fibres. Unexpectedly, a number of fibres showed localised COX-deficiency, with different regions of the fibre showing varying degrees of biochemical deficiency. The patient's homogenate skeletal muscle surprisingly had a lower mutation load than that measured in individual COX-positive fibres, possibly explained by contamination of the homogenate by the extensive fatty infiltrates observed in the biopsy.

The heteroplasmic m.3291T>C mutation in the T-Loop of mt-tRNA^Leu(UUR)^ (Fig. 3C) was identified by whole mtDNA sequencing of skeletal muscle from the patient, whilst maternal transmission was suggested through the sequencing of blood and urine from his unaffected sister. This mutation has been associated with a variety of mitochondrial disorders in several unrelated patients [2,5–7], however these reports have yet to provide conclusive proof of pathogenicity through single-fibre or *trans*mitochondrial cybrid studies. This lack of supportive data raised doubts regarding the role of this mutation in causing disease and evidence based primarily on the conserved nature of the affected position has proved insufficient to allay these concerns [8].

As has been observed in a number of cases, including most notably m.8344A>G [20], pathogenicity is not dependent upon the conservation or otherwise of the affected base. Although a useful measure of pathogenicity, as indicated by the fact that over 90% of definitely pathogenic mt-tRNA point mutations affect well-conserved positions, an over-emphasis on evolutionary conservation, compounded by an inconsistent selection of species for comparison means that relying on evolutionary conservation to classify mt-tRNA point mutations as pathogenic is, in the absence of supporting functional evidence, flawed [21,22].

The data presented here confirms the role of the m.3291T>C mutation in mitochondrial pathogenesis based on the revised mt-tRNA point mutation pathogenicity scoring system [21]. The mutation has been reported on several occasions, is heteroplasmic, segregates with disease and has both histochemical and biochemical evidences of respiratory chain deficiency. The mutation only shows moderate conservation (Fig. 3D) according to the consensus panel of species [22], but crucially, this report provides evidence from single-fibre studies that the mutation segregates with biochemical deficiency. Consequently, this mutation now scores 14 points according to the revised scoring system, and can be classified as ‘definitely pathogenic’ [21].

This investigation has provided conclusive evidence that supports the pathogenic role of the previously reported m.3291T>C mutation in human mitochondrial disease. Functional studies are essential for confirming the pathogenicity of mt-tRNA point mutations, and although indicative, the evolutionary conservation of affected positions should not be over-valued.

## Conflict of interest

The authors report no conflicts of interest.
